# Prevalence of* Toxoplasma gondii* and Other Gastrointestinal Parasites in Domestic Cats from Households in Thika Region, Kenya

**DOI:** 10.1155/2017/7615810

**Published:** 2017-06-13

**Authors:** Adele Nyambura Njuguna, John Maina Kagira, Simon Muturi Karanja, Maina Ngotho, Lucy Mutharia, Naomi Wangari Maina

**Affiliations:** ^1^Department of Biochemistry, Jomo Kenyatta University of Agriculture and Technology (JKUAT), P.O. Box 62000-00200, Nairobi, Kenya; ^2^Department of Animal Sciences, JKUAT, P.O. Box 62000-00200, Nairobi, Kenya; ^3^Department of Public and Community Health, JKUAT, P.O. Box 62000-00200, Nairobi, Kenya; ^4^Department of Animal Health & Production, Mount Kenya University, P.O. Box 342-01000, Thika, Kenya; ^5^Department of Molecular and Cellular Biology, Guelph University, 488 Gordon Street, Science Complex, Guelph, ON, Canada

## Abstract

Gastrointestinal (GIT) parasites of domestic cats (Felis catus) not only cause morbidity but are also potential zoonotic agents. The current study aimed at establishing the prevalence of GIT parasites in cats kept by households in Thika region, Kenya. Fecal samples were collected randomly from 103 cats and analyzed for presence of parasites using standard parasitological methods. In descending order, the prevalence of the detected protozoa parasites was* Isospora* spp. 43.7% (95% CI: 40.4–47%),* Cryptosporidium* spp. 40.8% (95% CI: 37.5–44.1%),* Toxoplasma gondii* 7.8% (95% CI: 4.5–11.1%), and* Entamoeba* spp. 2.9% (95% CI: 1.6–6.2%). The prevalence of the observed helminths was* Strongyloides stercoralis* 43.7% (95% CI: 40.4–47%),* Toxocara cati* 23.3% (95% CI: 20–26.6%),* Ancylostoma* spp. 9.7% (95% CI: 6.4–13%),* Dipylidium caninum* 8.7% (95% CI: 5.4–12.0%), and* Acanthocephala* spp. 1.9% (95% CI: 1–4.2%). The percentage of cats excreting at least one species of parasite was 73.2% (95% CI = 69.9–76.5%). The study shows that the cats have high spectrum (9) of parasites which are known to affect the cat's health and some are of zoonotic significance.

## 1. Introduction

Domestic cats* (Felis catus)* are important companions in many households, where they are mainly regarded as pets. In developing countries like Kenya, besides being pets, the cats are used to control rodents within and outside the households [1]. In spite of their importance, there are well-documented health hazards associated with owning cats [2]. There are a diverse range of infections, including parasitic, bacterial, fungal, and viral diseases that can be transmitted to humans from domestic cats. In absence of veterinary care, domesticated free roaming cats can cause public health problems and animal-welfare concerns in many countries [2]. Indeed, most of the diseases can be controlled or prevented by the owners of the cats if they are knowledgeable enough and have the resources to do so.

Gastrointestinal parasites of cats in Kenya and other African countries have received little attention as disease agents or as potential causes of zoonotic human diseases. The epidemiology of cat's parasites is affected by various factors including geographical region, presence of veterinary care, habits of the local animal populations, and the season of the year [3]. Epidemiological surveillance studies reported in various countries show that owned cats that are allowed to roam outdoors have high frequency of parasites [2–4]. The intestinal protozoa and helminth parasites of cats that have been identified by studies in other countries include* Giardia lamblia, Isospora* spp., hook worms, acanthocephalans,* Toxocara* spp.,* Physaloptera *spp.,* Taenia *spp.,* Joyexiella *spp.,* Dipylidium *spp.,* Dicrocoelium dendriticum *spp.,* Sarcocystis *spp.,* Entamoeba *spp., and* Blastocystis *spp. [2–4]. The parasites not only cause diseases in cats but are of zoonotic significance [3]. The fecal oral route is significant in the transmission of parasitic infections to humans especially in developing countries where poor personal hygiene is common [1]. Thus, poor disposal of litter from cats in such countries can contribute to transmission of zoonotic parasites.

In Kenya, one of the least studied zoonoses is toxoplasmosis which is an important water and food borne disease spread by* Toxoplasma gondii* whose main definitive host is the domestic cat. Oocysts from infected felids on excretion are unsporulated and are subspherical to spherical measuring 10–13 *µ*m in length and 0.5 *µ*m thick, whereas sporulated oocysts are subspherical to ellipsoidal measuring 11–14 *µ*m in length. Sporulated oocysts contain two ellipsoidal sporocysts with four sporozoites in each sporocyst. The oocysts, tachyzoites, and tissue cysts are the infective stages in the life cycle of* T. gondii. *All warm blooded animals, including man, can serve as intermediate hosts with tachyzoites developing as acute toxoplasmosis; the tachyzoites are then transformed to tissue cysts that are packed with many bradyzoites in them found in chronic toxoplasmosis. Tissue cysts are more prevalent in muscular and neural tissue but can be found in the other tissues where they remain latent and asymptomatic but toxoplasmosis can manifest in immunosuppression cases such as HIV [5].

In a recent study in Kenya, most of the homesteads were found to have cats which were used to control rodents within and outside the houses [1]. Further, only 2.8% of the population provided a litter box for the cats and the disposal of the litter was usually done on open ground increasing the risk of* Toxoplasma* oocysts and other intestinal parasites to human beings [1]. In Thika region of Kenya, toxoplasmosis has been shown to be prevalent in high risk groups, such as free range chicken slaughterhouse workers [6]. Although the abattoir workers most likely get toxoplasmosis from handling of the chicken meat, it is important to note that the birds obtain the infection from the environment contaminated by oocysts from cats [7]. The objective of the present study was to determine the prevalence of gastrointestinal (GIT) parasites present in household cats in Thika region, Kenya. The study contributes to the knowledge on GIT parasites of domestic cats and the findings will be used to create awareness on measures which can be used to prevent and control cat parasites in the study area.

## 2. Materials and Methods

### 2.1. Study Area and Climate

The study was carried out in Thika region; (latitude 1° 4′ 60 S 37°, longitude 4′ 60 E) located in Kiambu county, Central Kenya, in and covering 1960.2 km^2^. The region has a tropical climate with an annual rainfall ranging between 500 mm and 1500 mm while the mean temperature is 19.8°C. Rainfall is bimodal and long rains occur from mid-March to June while the short rains occur from mid-October to December. The mainstay for the economy is agriculture, majority of farmers in the Thika region being smallholders, practicing mixed agriculture, including livestock production, food, and cash crops [8]. Most of the families in the region have cats which are kept as pets and for controlling rodents [1]. In Thika region, the samples collected were from Ruiru, Juja, and Thika subcounties, which have urban and periurban set-up.

### 2.2. Fecal Collection and Examination

A cross-sectional study was undertaken in Thika region between January and March, 2015. Since the study focused more on toxoplasmosis, the sample size was calculated using the formula described previously [9]. In the formula, the expected prevalence of* T. gondii* for household cats was <7% [10] and thus a minimum of 100 cats were to be sampled. The cats in the current study were regarded as owned and lived both indoors and outdoors at different times of the day. The cats were fed household leftovers and slaughterhouse offal and were allowed to roam in the neighborhoods where they could feed on rodents and birds [1]. Following the development of a sampling frame of homesteads with cats in the study area, 110 households were randomly selected. Out of these households, 103 agreed to participate in the study. In this study, a total of adult 103 domestic cats aged ≥ 1 year were provided with litter boxes, from where the cats* (Felis catus)* defecated and fecal samples were collected thereafter. From each home a single sample of cat feces was obtained.

The fresh sample was put into a fecal collection pot, put into cool box, and transported to the laboratory at Jomo Kenyatta University of Agriculture and Technology where the feces were stored at +4°C and examined within 72 hours. All the 103 cat samples were tested for all the parasites. Each fecal sample was divided into two portions, without mixing or diluting. The first aliquot was assessed using formalin ether sedimentation technique [11], while the second aliquot was used to determine helminth eggs per gram of feces using the McMaster method [12].

### 2.3. Identification of Acid Fast Parasites

From the fresh fecal sample, (from first aliquot), smears were prepared and an acid fast staining method was undertaken using Kinyoun's carbol fuchsin and methylene blue stains as previously described [13]. Examination of 200 to 300 microscopic fields using 40x or higher objectives was then undertaken. For quality control, a control slide of* Cryptosporidium* spp. from a 10% formalin preserved specimen was included with each staining run.

### 2.4. Mouse Bioassay for* T. gondii*

*Toxoplasma gondii* was further identified using the mouse bioassay [14]. Fourteen (14) BALB/c mice used for the mouse bioassay were obtained from Institute of Primate and Research (IPR)-Nairobi, rodent breeding colony. They were housed in standard shoe box macron cages and were provided with bedding (wood shavings) and tunnels for burrowing. The mice were fed with mice pellets and provided water ad libitum.

Only samples which had* T. gondii* oocysts (see the formol ether method above) were evaluated. The oocysts were floated and harvested using sugar flotation technique [14]. They were thereafter washed in phosphate buffer saline appropriate dilutions until a desired count was achieved [14]. BALB/c male mice were infected orally with 10^4^ sporulated oocysts as previously described [15]. For each sample from an infected cat, one mouse was used. The mice were monitored for eight weeks after which they were euthanized using concentrated carbon dioxide; brain tissue was obtained and observed under the microscope for tissue cysts as previously described [14].

### 2.5. Polymerase Chain Reaction for* T. gondii*

A polymerase chain reaction (PCR) was undertaken on the brain samples from mice as previously described [7]. This process involved extraction of DNA using commercial DNA extraction kit (Zymo Research Quick-gDNA® Miniprep Kit, USA). Nested PCR reaction targeting a repetitive 529 bp DNA fragment sequence (GenBank Accession number AF146527) was performed as previously described [16]. The reference* T. gondii* (RH) DNA was used as positive control, while PCR water was used as the negative control. The product generated in the second amplification was run in 1.5% agarose gel prestained with 3 *μ*L of ethidium bromide (1 *μ*g/mL) and visualized under ultraviolet light.

### 2.6. Ethical Clearance

Ethical clearance for the studies involving animals was approved by Institutional Animal Care and Use Committee of the Institute of Primate Research, Nairobi (Ethical clearance certificate IRC/21/11). The study also adhered to the ARRIVE guidelines for reporting in vivo animal experiments.

### 2.7. Data Analysis

Data was entered into Microsoft Excel (Microsoft, USA) and exported to SPSS Version 16.0 (SPSS, Inc., Chicago, IL, USA) for analysis. Prevalence was determined as the number of infected cats divided by number of sampled cats. Data was presented in form of a figure and table.

## 3. Results

**Figure 1:**
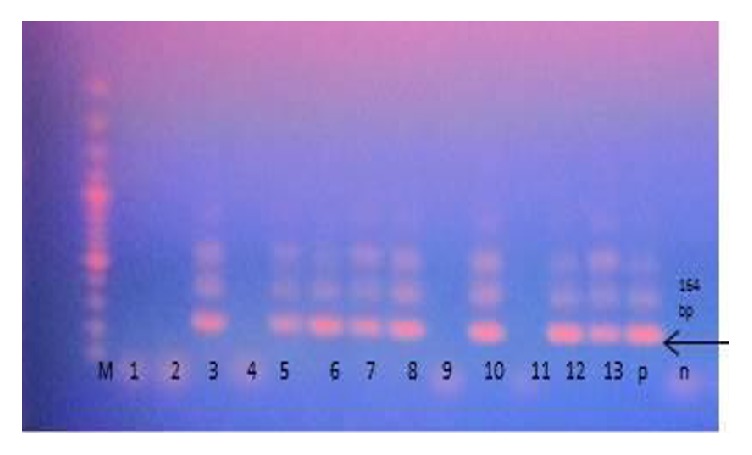
Gel photograph for secondary PCR amplification products (164 bp) of* T. gondii* in brain tissues of mice infected with oocysts cats from Thika region, Kenya. Lanes 3, 5, 6, 7, 8, 10, 12, and 13 are positive. M: 100 bp DNA ladder, p: positive control, and n: negative control.

Out of the 103 cat fecal samples, nine GIT parasites were identified. Four protozoan parasites with a prevalence ranging from 2.9% to 43.7% were observed in the study and their magnitudes in descending order were* Isospora* spp.,* Cryptosporidium* spp.,* Toxoplasma gondii,* and* Entamoeba* spp. (Table 1). Thirteen cats (12.6%) were found to be excreting oocysts which had similar morphology to those of Toxoplasma. Since other parasites such as Hammondia have similar morphology to Toxoplasma, the presence of* T. gondii* was confirmed by the mouse bioassay and PCR. Eight brain samples from the mice were positive of* T. gondii* DNA (Figure 1). Thus, the prevalence of* T. gondii* in the cats was 7.8% (8/103).

Five helminths observed in the study included nematodes and cestodes (Table 1). The prevalence of the helminthes ranged from 1.9% to 43.7%.* Strongyloides stercoralis *accounted for a highest prevalence (43.7%) of all the intestinal nematodes excreted by the cats. Both larvae and eggs of* S. stercoralis *were observed. Other eggs of helminths observed in the cat feces in descending order included* Toxocara cati, *hook worms (*Ancylostoma* spp.),* Dipylidium caninum, *and* Acanthocephalan* spp.

The percentage of cats excreting at least one species of parasite was 73.2% (95% CI = 69.9–76.5). A substantial proportion (33%) of the cats had monoinfections of a given parasites species mainly* S. stercoralis* and* Isospora* spp. (Table 2). Mixed infection parasites (40.2%) ranging from two to five parasite species were found in cats feces. The parasite spectrum showed a skewed binomial distribution.

## 4. Discussion

In most rural and urban Kenya, domesticated cats are mainly kept for rats control and to deter snakes and other vermin from the homesteads [1]. The owners do not deworm these cats and veterinary care is minimal if not absent. Further, the disposal of feces from these cats is wanting and predisposes human beings to variety of zoonotic parasites [1]. The current study is the first one to report on occurrence of gastrointestinal parasites in cats in Kenya. The present study shows a high prevalence of both helminth and protozoan parasites. Studies done in different countries indicate high prevalence of intestinal parasites. For example prevalence ranging from 41% to 91% has been reported in northern region in Egypt and China [3, 4]. The high occurrence of intestinal parasites in cat in the study area could be due to lack of veterinary care where owners deworm food producing animals but rarely is it done for the dogs and cats [1]. The disposal of cat feces could also be contributing to high prevalence of these parasites; the owners rarely provide litter boxes for defecating, with disposal of the waste being mainly in the garden. This leads to increased exposure to farmers since they use bare hands when doing most of the farm practices and rarely wash their hands before eating [1, 17].

All the parasites observed in the current study can affect cats' health and are of zoonotic significance.* Strongyloides stercoralis* was the most important nematode and prevalence reported was higher than reported in other countries [18]. Apart from causing diarrhea in cats, Strongyloidiasis causes severe morbidity in human beings and the global human prevalence of is estimated at between 30 and 100 million infected persons, mostly in developing countries. In humans, the most likely way of becoming infected with Strongyloides is from soil contaminated with Strongyloides larvae which penetrates barefooted people. Strongyloidiasis infection in human beings can be severe and life-threatening in immunosuppressed patients [19]. It would be important to ascertain possible transmission of Strongyloides between the human and cat populations in the study area.

*Toxocara cati* prevalence reported in this study was higher than that reported in China [3]. In heavy infections,* Toxocara cati* causes toxocariasis in cats but light infections are asymptomatic. However, if the parasite infects the human beings, the parasite may cause ocular and visceral larva migrans [4]. Prevalence of toxocariasis has not been widely reported in Africa, and the current study should inform further studies in the human populations.

The prevalence of hookworms in this study was higher than those reported for well-kept cats [20, 21]. Lack of deworming of cats in the present study could have led to the high prevalence. The main hookworm's species affecting cats include* Ancylostoma tubaeforme*,* A. braziliense,* and* Uncinaria stenocephala*, although the latter is quite rare. Hookworms suck blood in cats leading to development of anemia and diarrhea. In humans, contact with affected soil can lead to the development of cutaneous larva migrans, although such cases are rarely recorded in Kenya [22].

The prevalence of the coccidian parasites* Cryptosporidium *spp.,* Isospora,* and* T. gondii* was quite high. For* T. gondii, *the prevalence reported in this study was higher than that reported in cats in Bahrain and Sudan [23] but lower than that reported in Ethiopia [24]. The presence of risk factors for toxoplasmosis was recently documented in Thika region [1]. In the latter study, households kept cats as pets and controlling rodents which could be one of the sources of* T. gondii* infections in the cats. Others source of* Toxoplasma* bradyzoites could be practice of feeding raw animal offals to the cats, which is common in the study region [1]. Recent studies in Thika region showed that up to 39% of the slaughterhouse workers and 79% of free range chicken were infected with* T. gondii* [6, 7]. It would be important to carry out an epidemiological study to determine the relationship between the occurrence of toxoplasmosis in the cats and human beings and various risk factors in the study area. In the current study, five of the thirteen Toxoplasma-like oocysts which could not be identified by PCR could have been* Hammondia* species which do not cause clinical disease in cats [25].

*Isospora* species which causes clinical coccidiosis in cats are mainly* Isospora felis* and* Isospora rivolta*. The prevalence reported in this study is higher than that reported in China [3]. In cats, coccidiosis is associated with other infectious agents, immunosuppression, or stress. In immunocompetent people, isosporiasis is asymptomatic but is pathological in immune suppressed patient [26]. The prevalence of* Cryptosporidium *spp. in the present study was higher than that reported in other studies [27]. Similar to other parasites reported above, the high prevalence could be attributed to fecal oral transmission, where the oocysts are infective at excretion. There are a number of zoonotic species including* C. parvum* and C*. felis* which can be transmitted from cats to human beings and these have been reported in Kenya [28]. The parasite rarely causes disease in immunocompetent hosts but is a leading protozoa cause of diarrhea in children worldwide and is a common cause of outbreaks in child-care centers. In immunodeficient individuals the symptoms are severe with profuse diarrhea and can be fatal [29].

## 5. Conclusion

Zoonotic gastrointestinal parasites are a major public health concern and have been recognized by World Health Organization as neglected tropical diseases that significantly affect livelihoods of resource poor people in developing countries. The cat being a domestic animal with close contact with human beings plays a crucial role in transmission of these parasites. The current study has identified 9 species of parasites which can cause important disease in cats and are of zoonotic importance. The study recommends that the animal health workers in the study area should create awareness of the existence of the parasites and advocate for effective control strategies such as regular deworming, proper feeding, and keeping of cats indoors and proper disposal of cat feces. Further, there is need to determine the disease epidemiology of these parasites and characterizing the transmission patterns between cats and humans in Kenya and other developing countries is of paramount importance. Such information will be important in creating awareness of the important parasitic infections in cats among the local human population and health and veterinary professionals with an intention of curtailing transmission of the parasites.

## Figures and Tables

**Table 1:** Prevalence and intensity of gastrointestinal parasites found in 103 cats in Thika region, Kenya.

	Number of infected cats	Prevalence (%) 95% CI	Mean EPG
*Protozoa*			
*Isospora *spp.	45	43.7 (40.4–47)	ND
*Cryptosporidium *spp.	42	40.8 (37.5–44.1)	ND
*Toxoplasma gondii*	8	7.8 (4.5–11.1)	ND
*Entamoeba *spp.	3	2.9 (1.5–6.2)	ND
^*∗*^Others	5	4.9 (1.5–8.2)	ND
*Helminths*			
*Strongyloides stercoralis*	45	43.7 (40.4–47)	638
*Toxocara cati*	24	23.3 (20–26.6)	1027
Hookworms (*Ancylostoma *spp.)	10	9.7 (6.4–13)	417
*Dipylidium caninum*	9	8.7 (5.4–12)	389
*Acanthocephalan *spp.	2	1.9 (1–4.2)	800

EPG: eggs per gram of faeces, ND: not done, ^*∗*^others: toxoplasma-like oocysts (*T. gondii* negative by PCR) of uncertain aetiology, and 95%: 95% confidence interval.

**Table 2:** Number of gastrointestinal parasites species found in 103 cats from Thika region, Kenya.

Number of parasite species per cat	Number infected	% of infected cats
One species	34	33
Two species	21	20
Three species	14	13.5
Four species	6	5.8
Five species	1	0.9
No infection	—	26.8
